# Machine Learning and Deep Learning Frameworks for Human–Virus Protein–Protein Interaction Prediction: Emerging Architectures, Methods, Benchmarks, and Challenges

**DOI:** 10.3390/ijms27136034

**Published:** 2026-07-05

**Authors:** Subhadeep Basu, Dipanwita Adhikary, Kuntal Ghosh, Swarup Chattopadhyay, Shramana Deb, Ritwick Mondal, Jayanta Roy, Anjan Chowdhury, Julián Benito-León

**Affiliations:** 1 Department of Biotechnology, Amity University, Noida 201313, India; subhadeepbasu1255@gmail.com; 2 Department of Biotechnology and Biochemical Engineering, Indian Institute of Technology, Kharagpur 721302, India; dadhikari412@gmail.com; 3 Machine Intelligence Unit, Indian Statistical Institute, Kolkata 700108, India; kuntalghos@gmail.com; 4 School of Computer Science and Engineering, XIM University, Bhubaneswar 751013, India; swarupchatt@gmail.com; 5 Centre for Neurovascular Research, Manipal Group of Hospitals, Kolkata 700099, India; shramanadeb1995@gmail.com (S.D.); ritwickraw@gmail.com (R.M.); jroyneuro01@gmail.com (J.R.); 6 Department of Neurology, Manipal Group of Hospitals, Kolkata 700099, India; 7 Department of Computer Science and Engineering, Indian Institute of Technology, Dhanbad 826004, India; 8 Department of Neurology, 12 de Octubre University Hospital, 28041 Madrid, Spain; 9 Group of Neurodegenerative Diseases, Hospital Universitario 12 de Octubre Research Institute (Imas12), 28041 Madrid, Spain; 10 Network Center for Biomedical Research in Neurodegenerative Diseases (CIBERNED), 28031 Madrid, Spain; 11 Department of Medicine, Faculty of Medicine, Complutense University of Madrid, 28040 Madrid, Spain

**Keywords:** protein–protein interaction (PPI), human–virus interaction, network prediction, biological databases, computational models, machine learning, deep learning, graph neural networks

## Abstract

The outbreak of coronavirus disease 2019 (COVID-19), caused by severe acute respiratory syndrome coronavirus 2 (SARS-CoV-2), has emerged as one of the most significant global health crises in recent history. Coronaviruses are a diverse group of RNA viruses classified into alpha, beta, gamma, and delta genera, with SARS-CoV-2 belonging to the beta-coronavirus family. The virus exhibits high transmissibility and causes a wide spectrum of clinical manifestations ranging from mild respiratory symptoms to severe complications such as acute respiratory distress syndrome, multi-organ failure, and death, particularly among elderly and immunocompromised individuals. Structurally, SARS-CoV-2 possesses a large single-stranded RNA genome encoding major structural proteins, including spike (S), envelope (E), membrane (M), and nucleocapsid (N) proteins, which play critical roles in host-cell recognition and viral infection. Understanding the molecular mechanisms of virus–host interactions, especially protein–protein interactions (PPIs), is essential for uncovering viral pathogenesis and identifying potential therapeutic targets. Traditional experimental techniques for PPI detection, such as yeast two-hybrid and affinity purification methods, are often expensive, labor-intensive, and prone to inaccuracies. Consequently, computational approaches based on machine learning (ML) and deep learning (DL) have gained significant attention for efficient and scalable PPI prediction. These methods use diverse biological information, including protein sequences, structural features, genomic data, Gene Ontology annotations, and interaction networks, to model complex biological relationships. This survey reviews computational approaches to PPI prediction, highlighting ML- and DL-based techniques, methodological advances, performance evaluation practices, and limitations that affect benchmark comparability. It also discusses biological databases and data sources commonly used in PPI studies and explicitly considers how models trained in coronavirus-centered settings may generalize to other viral families with different mechanisms of host interaction.

## 1. Introduction

The emergence of novel zoonotic pathogens, exemplified by the family Coronaviridae, underscores a persistent threat to global public health, economic stability, and healthcare infrastructure [1,2,3,4,5]. Coronaviruses are categorized into four genera, with alpha- and beta-coronaviruses predominantly infecting mammalian hosts, and have historically triggered severe epidemics, including the SARS outbreak in 2003 [6,7] and the MERS outbreak in 2012 [8], culminating in the global crisis of SARS-CoV-2. These single-stranded RNA viruses possess exceptionally large genomes of approximately 27–32 kb [9] and encode four primary structural proteins—spike (S), envelope (E), membrane (M), and nucleocapsid (N)—which contribute to viral pathogenesis and severe clinical manifestations ranging from acute respiratory distress to systemic multi-organ failure [5,10]. Host-cell invasion is mediated by the physical interaction between the viral spike glycoprotein receptor-binding domain (RBD) and specific host-cell surface receptors, such as DPP4 for MERS-CoV and ACE2 for SARS-CoV and SARS-CoV-2 [11,12,13]. Following cellular entry, viruses exploit host machinery through virus–host protein–protein interactions (PPIs) to facilitate replication, translation, immune evasion, and host-cell remodeling. While high-throughput experimental methodologies such as yeast two-hybrid screening, affinity purification, tandem affinity purification, and protein microarrays remain foundational for mapping interactomes, they are constrained by experimental cost, prolonged timelines, and false-positive and false-negative findings. Computational frameworks based on ML and DL, therefore, provide rapid, scalable, and high-throughput alternatives that leverage primary sequences, three-dimensional structural configurations, Gene Ontology annotations, and genomic networks. These in silico approaches broadly include traditional ML paradigms based on manual feature engineering and modern DL models, including sequential non-graph architectures and geometry-aware graph neural networks. Given the rapid evolution of viral genomes and the large number of proposed computational frameworks, there is a need for a structured synthesis of predictive pipelines, benchmark designs, and generalization constraints. This review provides a focused analysis of emerging ML and DL frameworks for human–virus PPI prediction, complements broader surveys of computational PPI prediction by emphasizing virus–host systems, and synthesizes reported evaluations rather than presenting a new original benchmark experiment.

## 2. Literature Search Strategy

This review was conducted using a structured, semi-systematic literature framework to comprehensively synthesize contemporary computational approaches to predicting human–virus PPIs. Given the rapidly evolving, interdisciplinary intersection of computational interactomics, structural bioinformatics, artificial intelligence (AI), and graph representation learning, a semi-systematic review was chosen over a strictly systematic design to enable a broader conceptual integration of emerging algorithmic paradigms and methodological innovations across heterogeneous sources of literature.

A comprehensive electronic literature search was conducted across multiple scientific databases: PubMed/MEDLINE, Scopus, Web of Science Core Collection, IEEE Xplore Digital Library, and Google Scholar. The search targeted studies published between January 2005 and March 2025, thereby encompassing both foundational machine learning-based PPI prediction frameworks and contemporary transformer-based or graph neural network architectures developed during the modern deep learning era. The final database search was completed in November 2025, and English-language scholarly records with sufficient methodological relevance and accessible full texts were considered for inclusion.

PubMed/MEDLINE served as the principal biomedical retrieval platform due to its integration with the National Library of Medicine (NLM) Medical Subject Headings (MeSH) indexing system. To maximize retrieval precision and semantic coverage, the search strategy combined controlled MeSH vocabulary terms with free-text keyword expansion, Boolean retrieval operators, phrase searching, truncation, and semantic synonym mapping. MeSH descriptors were selected according to their direct relevance to protein interaction biology, host–pathogen systems, structural bioinformatics, AI, graph representation learning, and computational biology.

The principal MeSH descriptors incorporated into the search framework included “Protein Interaction Mapping,” “Protein Binding,” “Host-Pathogen Interactions,” “Viruses,” “Machine Learning,” “Deep Learning,” “Artificial Intelligence,” “Algorithms,” “Neural Networks, Computer,” “Sequence Analysis, Protein,” “Protein Structure, Tertiary,” “Computational Biology,” “Data Mining,” “Graph Theory,” and “Systems Biology.” These controlled vocabulary descriptors were supplemented with free-text terms including “protein–protein interaction,” “human–virus interactome,” “host–virus interaction,” “graph neural network,” “geometry-aware learning,” “protein language model,” “transformer architecture,” “deep representation learning,” “graph attention network,” and “structure-aware PPI prediction” to improve sensitivity for recently emerging computational methodologies that may not yet be fully indexed within conventional MeSH hierarchies. Boolean retrieval logic was implemented using a concept-block search architecture in which semantically related terms were linked with the OR operator, whereas biologically distinct conceptual domains were linked with the AND operator.

### 2.1. Eligibility Criteria and Study Selection

The screening pipeline strictly enforced clear boundaries to ensure both methodological relevance and data transparency. Studies were considered eligible based on the following explicit criteria: The study must describe computational methodologies for predicting protein–protein interactions in human–virus or host–pathogen systems. The predictive framework must incorporate machine learning, deep learning, graph representation learning, probabilistic modeling, transformer architectures, or hybrid computational systems. The manuscript must report benchmark transparency by detailing the explicit dataset construction methodology, negative sampling strategies, external validation procedures, and evaluation metrics. The reported performance evaluation must prioritize robust metrics such as precision–recall area under the curve (PR-AUC), the Matthews correlation coefficient (MCC), F1-score, or independent test performance to guard against the artificial inflation of accuracy that is typical of highly imbalanced PPI datasets.

Conversely, records were systematically excluded from the evidence synthesis based on the following rules: studies exclusively focused on wet-laboratory experimental interaction detection without any computational modeling component were omitted; editorials, commentary pieces, non-scholarly reports, and duplicate publications were excluded; and conference abstracts lacking methodological transparency, architectural detail, or an accessible full text were removed from the selection pool. Retrieved records underwent sequential title screening, abstract evaluation, and full-text assessment. Duplicate records identified across multiple databases were removed using a combination of automated and manual deduplication procedures. During full-text assessment, studies were critically evaluated according to computational novelty, reproducibility, biological applicability, and architectural significance. Additional backward and forward citation tracking was conducted for highly influential publications to identify relevant studies not captured during the primary database retrieval process.

### 2.2. Data Extraction and Evidence Synthesis

For each eligible study, structured data extraction was performed to systematically collect information regarding biological dataset source, organism or viral system, feature representation modality, computational architecture, graph construction strategy, benchmark design, evaluation metrics, validation protocol, and predictive performance. Particular attention was paid to studies employing graph neural networks, protein language models, transformer-based architectures, multimodal feature integration, explainable AI frameworks, and structure-aware learning systems, given their growing importance in modern computational interactomics.

The extracted studies were subsequently categorized into major computational paradigms, including feature-based ML approaches, network embedding frameworks, non-geometry-aware DL architectures, geometry-aware graph neural networks, temporal and dynamic PPIN models, heterogeneous graph learning systems, probabilistic computational models, and structure-aware residue-level learning frameworks. Comparative synthesis focused on identifying methodological trends, architectural strengths and limitations, benchmark heterogeneity, interpretability challenges, scalability constraints, and emerging research directions in AI-driven protein interaction prediction.

Because of substantial heterogeneity across datasets, benchmark construction procedures, negative sampling strategies, validation protocols, and evaluation metrics, quantitative meta-analysis was not performed. Consequently, the evidence synthesis presented in this review is qualitative and conceptual in nature, with comparative analyses intended to provide methodological interpretation and architectural insight rather than strict cross-study performance ranking. Studies lacking sufficient methodological transparency in dataset construction, negative sampling procedures, or validation strategies were interpreted with caution during comparative synthesis to minimize overestimation of reported predictive performance.

To improve transparency, the present synthesis distinguishes qualitative evidence mapping from quantitative meta-analysis. The review therefore emphasizes benchmark design, split protocols, negative sampling, and external validation as major determinants of reported performance, rather than treating reported accuracy values as directly comparable across heterogeneous studies.

## 3. Biological Databases and Data Modalities for PPI Prediction

**Figure 1:**
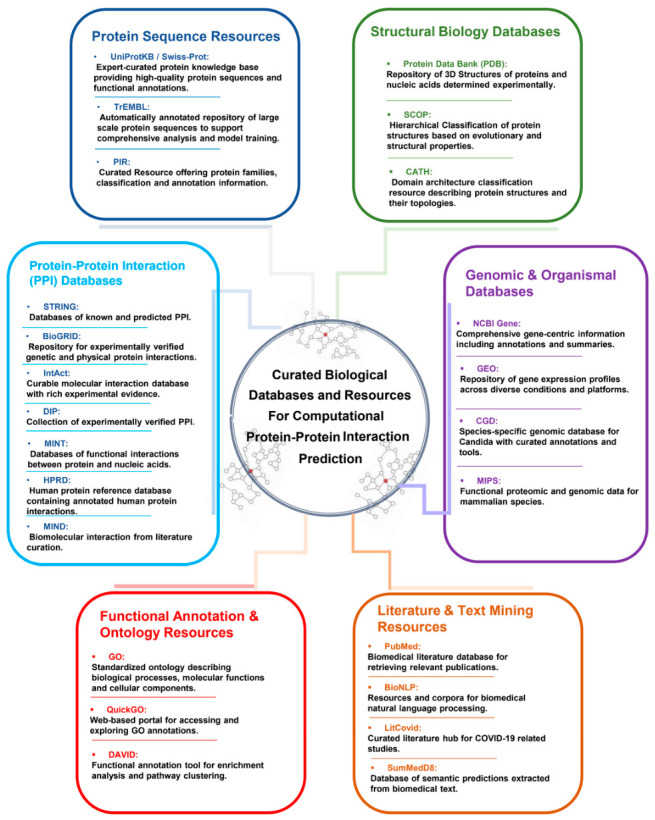
Biological databases and multimodal data resources used in computational protein–protein interaction (PPI) prediction. The illustration summarizes the major categories of biological databases integrated into modern PPI prediction pipelines, including sequence repositories, structural databases, interaction resources, genomic/transcriptomic datasets, functional annotation systems, and literature/text-mining platforms. The figure further highlights the data harmonization and integration workflow used to generate biologically relevant, high-confidence PPI networks for downstream computational and systems biology applications. Abbreviations: PPI, protein–protein interaction; GO, Gene Ontology; PDB, Protein Data Bank; DAVID; Database for Annotation Visualization and Integrated Discovery, Gene Expression Omnibus; HPIDB, Host–Pathogen Interaction Database; BioGRID, Biological General Repository for Interaction Datasets; MIND, Membrane-Protein Interaction Network Database; MINT, Molecular Interaction Database; IntAct, Interaction Action; HPRD, Human Protein Reference Database; DIP; Database of Interacting Proteins; CGD, Candida Genome Database; MIPS, Munich Information Center for Protein Sequences; CATH; Class Architecture Topology Homologous Superfamily; SCOP; Structural Classification of Proteins; PIR; Protein Information Resource.

The advent of high-throughput experimental technologies has generated an exponential increase in interactome data since 2005, transforming computational PPI prediction from early genome-reliant mapping into a big-data discipline. While this data deluge poses distinct computational scalability challenges, it simultaneously provides a rich substrate for deep learning architectures. Modern framework paradigms increasingly favor integrative strategies that synthesize diverse data modalities to decode complex interactome networks. These multi-modal pipelines leverage distinct protein descriptors across five primary data categories: primary sequences, higher-order structures, Gene Ontology (GO) annotations, genomic contexts, and topological network features (Figure 1, Table 1).

### 3.1. Sequence-Based Information

The primary structure of a protein, represented linearly by its amino acid sequence, serves as the baseline data modality for computational PPI prediction due to its massive volume and relative ease of acquisition. Because the primary sequence intrinsically dictates higher-order folding configurations, it serves as a robust standalone proxy for predictive modeling when structural or functional annotations are missing. To capture these interactions mathematically, computational pipelines extract sequence-derived descriptors that quantify pairwise similarities. These feature vectors typically encompass fundamental physicochemical properties—including hydrophobicity and amino acid composition—as well as evolutionary conservation profiles derived from multiple sequence alignments, such as Position-Specific Scoring Matrices (PSSMs) [14,15,16]. Such evolutionary conservation landscapes reveal critical co-evolutionary patterns essential for predicting stable binding interfaces [17]. Standardized repositories hosting these primary sequence datasets include the Protein Information Resource (PIR), UniProt [18], and SWISS-PROT [19].

### 3.2. Higher-Order Structural Data

Protein higher-order structures (HOSs) span hierarchical levels of organization, starting from local secondary structural motifs (α-helices and β-sheets) dictated by backbone hydrogen bonding, extending to three-dimensional tertiary conformations of single chains, and culminating in quaternary multimeric assemblies (e.g., dimers or higher-order oligomers). Guided by Anfinsen’s thermodynamic hypothesis, which states that a protein’s native three-dimensional structure is determined by its primary amino acid sequence [20], many computational architectures continue to prioritize sequence features. This choice is further reinforced by the stark data asymmetry in public repositories: experimentally resolved 3D structures are far scarcer than the abundance of raw sequence data, fundamentally limiting the scalability of purely structure-based models [21,22]. Nonetheless, when available, structural data provides precise geometric insights into spatial binding interfaces and thermodynamic binding affinities. Curated repositories mapping this structural landscape include the Structural Classification of Proteins (SCOP) [23], the Protein Data Bank (PDB) [24], and CATH [25].

### 3.3. Gene Ontology (GO) Annotations

The Gene Ontology (GO) consortium offers a highly structured, standardized framework to encapsulate functional, biological, and spatial knowledge of gene products across diverse organisms [26,27]. GO annotations are systematically partitioned into three distinct domains: molecular function (MF), which catalogs biochemical activities such as catalytic or binding mechanisms; biological process (BP), which charts broader multi-step pathways such as signal transduction cascades; and cellular component (CC), which determines precise macromolecular or subcellular localization. In PPI prediction, proteins operating within the same pathway or localized to the same cellular compartment exhibit a significantly higher statistical probability of physical interaction. Computational models capture this functional proximity by computing semantic similarity metrics across GO directed acyclic graphs, markedly improving overall classification accuracy [28,29]. These curated annotations are dynamically maintained and accessed via the GO database [27] and QuickGO [30].

### 3.4. Genomics-Based Features

Genomic features offer a deep evolutionary perspective on protein co-regulation and physical assembly, rooted firmly in the central dogma of molecular biology where DNA is transcribed into mRNA and subsequently translated into functional polypeptide chains [31]. Driven by advances in whole-genome sequencing, modern computational workflows exploit cross-species genomic conservation to infer physical interactions between translated proteins. Three primary genomics-based features serve as indicators of functional or physical linkage: gene fusion events, gene neighborhood conservation, and phylogenetic profiling [32,33]. Gene fusion occurs when independent genes in a reference organism are expressed as a single, contiguous open reading frame in another species, strongly implying cooperative function. Similarly, conserved gene neighborhoods (such as operons) denote spatial genomic proximity associated with co-expression, while phylogenetic profiles trace the correlated presence or absence of genes across evolutionary lineages, revealing shared functional networks. These evolutionary markers can be retrieved from specialized genomic repositories such as the Candida Genome Database [34] and the Munich Information Centre for Protein Sequences (MIPS) [35].

### 3.5. Network-Based Topologies (PPINs)

At the systems level, individual physical interactions are synthesized into comprehensive Protein–Protein Interaction Networks (PPINs), which provide a holistic framework for mapping complex cellular pathways, annotating uncharacterized proteins, and identifying critical disease hubs or therapeutic targets [36]. Formally, a PPIN is mathematically modeled as a graph,

(*G* = (*V*, *E*)),

where the vertex set V represents individual proteins and the edge set E denotes verified physical or functional interactions. This abstraction allows graph-based deep learning and machine learning algorithms to exploit both local topological features (such as node degree and centrality) and global network modularity. Comprehensive interactome repositories archiving these topological networks include STRING [37], BioGRID [38], BIND [39], DIP [40], MINT [41], HPRD [42], and IntAct [43]. Network visualization and exploratory analysis tools, such as Cytoscape [44], are best interpreted as software environments for integrating and visualizing biomolecular interaction networks, rather than as sequence-based ML classifiers for PPI prediction.

As a conceptual synthesis of this entire multimodal landscape, Figure 1 delineates the overarching predictive workflow, charting the seamless trajectory from primary database curation and multi-feature extraction to the execution of an integrative computational model and subsequent downstream PPIN topological analysis for target and mechanism discovery.

## 4. Machine Learning (ML)-Based Approaches for PPI Prediction 

**Figure 2:**
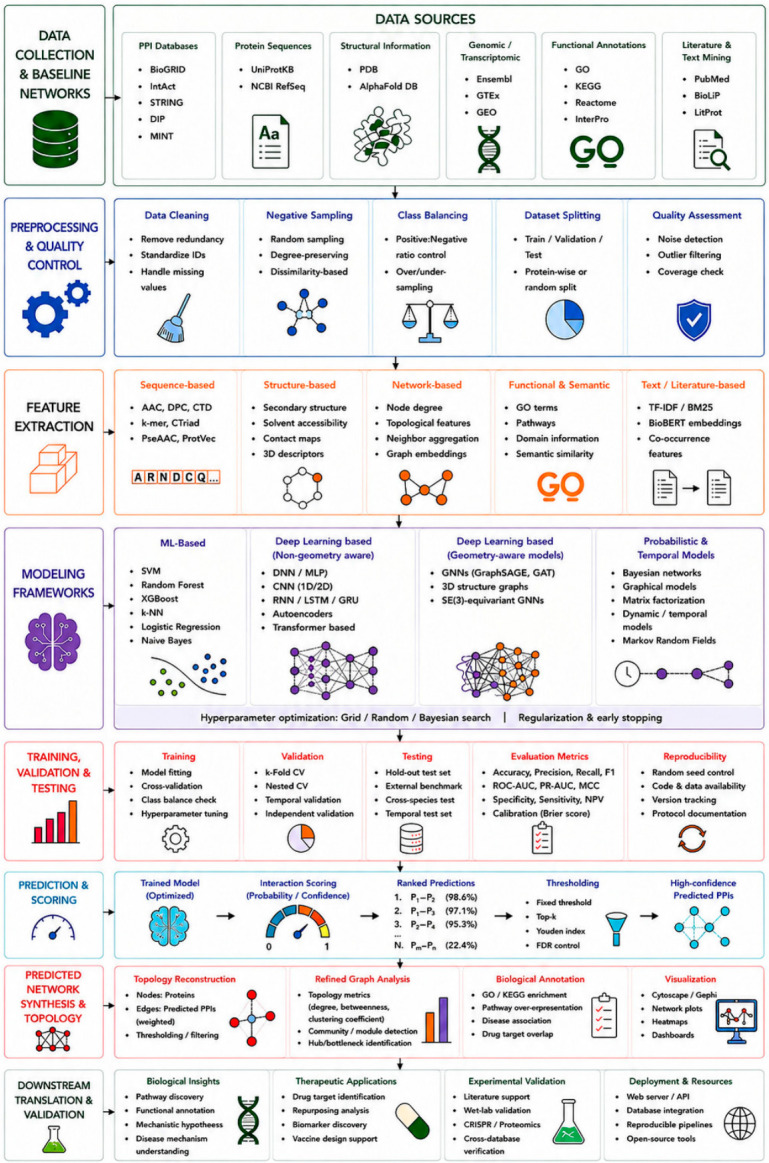
Analytical framework for efficient protein–protein interaction (PPI) network prediction integrating machine learning (ML), deep learning (DL), and Bayesian approaches. The schematic illustrates a unified computational workflow for human–virus PPI prediction, beginning with the acquisition of multi-source biological data from protein sequence repositories, structural databases, PPI databases, genomic/transcriptomic resources, functional annotation systems, and literature-derived datasets. Following preprocessing, feature extraction modules generate sequence-based descriptors, structural representations, network topology features, Gene Ontology (GO)-based semantic annotations, and text-derived embeddings for downstream predictive modeling. The ML-based framework employs engineered feature vectors and classifiers such as SVMs, RFs, Gradient Boosting, k-NNs, logistic regression, and Naïve Bayes. The DL-based framework integrates tensor, sequence, and graph-based representations via MLPs, CNNs, recurrent neural networks (RNNs/LSTMs/GRUs), graph neural networks (GNNs), graph attention networks (GATs), autoencoders, and attention-based architectures. Bayesian modules model interaction uncertainty using Bayesian logistic regression, Bayesian networks, Gaussian processes (GPs), Bayesian matrix factorization, and variational probabilistic learning approaches. Models are subsequently trained and validated using multiple evaluation metrics and cross-validation strategies before generating interaction probability scores. High-confidence predictions are assembled into weighted PPI networks for downstream network construction, topological analysis, hub and module identification, biological interpretation, therapeutic target prioritization, and systems-level interactome analysis. The framework highlights the integration of multimodal biological knowledge with advanced artificial intelligence paradigms for scalable and interpretable PPI network prediction. Abbreviations: PPI, Protein–Protein Interaction; PPIN, Protein–Protein Interaction Network; ML, Machine Learning; DL, Deep Learning; SVM, Support Vector Machine; RF, Random Forest; k-NN, k-Nearest Neighbor; MLP, Multi-Layer Perceptron; CNN, Convolutional Neural Network; RNN, Recurrent Neural Network; LSTM, Long Short-Term Memory; GRU, Gated Recurrent Unit; GNN, Graph Neural Network; GCN, Graph Convolutional Network; GAT, Graph Attention Network; GO, Gene Ontology; GP, Gaussian Process.

Driven by the deluge of high-throughput biological data, machine learning (ML) paradigms have revolutionized PPI prediction, offering robust computational toolsets for molecular biology and accelerated drug discovery. By leveraging large-scale datasets and advanced algorithms, ML models can accurately predict interactions and decipher complex relational networks. In this review, we categorize ML-based approaches into two primary paradigms: feature-based methods and network embedding-based frameworks (Table 2, Figure 2).

### 4.1. Feature-Based Approach

Feature-based ML represents the traditional machine learning paradigm, in which hand-crafted biological, physicochemical, or structural descriptors are mathematically extracted from protein data prior to training a downstream classifier or regressor. We outline the key classical feature-based classification techniques below.

#### 4.1.1. SVM-Based PPIN Classification

A Support Vector Machine (SVM) [67] is a supervised learning algorithm used for classification and regression that finds an optimal separating hyperplane with a maximum margin. It efficiently handles non-linear data by using the kernel trick to map inputs into a higher-dimensional feature space. SVM was used to predict human cellular targets of SARS-CoV-2 by combining primary sequence information, amino acid composition, pseudo-amino acid composition, and conjoint triad properties. Similarly, linear SVM classifiers have been optimized strictly using sequence-derived physicochemical property scales extracted from primary structures [51,60,63]. Early foundational work [14] demonstrated that an SVM trained with a conjoint triad feature description and an S-kernel function could accurately replicate primary interaction topologies across complex crossover networks.

To capture localized sequence patterns, an autocovariance (AC) encoding methodology combined with an SVM was proposed [45], wherein seven critical physicochemical indices—hydrophobicity, hydrophilicity, polarity, polarizability, side-chain volume, solvent-accessible surface area, and net side-chain charge—are numerically scaled across a 30-residue sliding window, achieving an 87.36% classification accuracy on *Saccharomyces cerevisiae*. In host–pathogen contexts, Dyer et al. [47] integrated domain profiles, sequence k-mer composition, and human protein properties within a linear SVM to map human–HIV interactions. Subsequent models used Conjoint Triad (CT) encodings paired with radial basis function (RBF) kernels to achieve superior predictive accuracy across human papillomavirus (HPV) and hepatitis C virus (HCV) datasets [48]. This sequence-only predictive capacity was further validated by Alguwaizani et al. [58], who leveraged basic amino acid compositions and repeat patterns within an SVM architecture to outperform contemporary state-of-the-art benchmarks [68,69].

#### 4.1.2. RF-Based PPIN Classification

Random Forest (RF) and its ensemble variants demonstrate high generalization capabilities across cross-species datasets. In recent studies, modern natural language processing (NLP)-driven embeddings such as doc2vec have been coupled with RF classifiers to achieve improved human–virus PPI prediction performance compared with legacy encoding schemes [61]. Similarly, combining Position-Specific Scoring Matrices (PSSMs) with a 400-dimensional discrete Hartley transform (DHT) descriptor within a Rotation Forest (RoF) classifier yielded superior predictive accuracy compared with standalone RF, SVM, k-NN, and AdaBoost models across human, yeast, and *Oryza sativa* proteomics [64]. To explicitly model bacterial pathogenesis, integrated predictors for human–*Yersinia pestis* PPIs have combined topological network properties (NetTP, NetSS) with multi-scale sequence encodings (CKSAAP, PseTC, AC) via an RF-driven noisy-OR ensemble, achieving a robust area under the receiver operating characteristic curve (AUC) of 0.922 [60].

#### 4.1.3. KNN-Based PPIN Classification

The k-Nearest Neighbor (k-NN) algorithm represents a simpler, non-parametric approach that remains valuable when paired with robust feature extraction techniques. Implementing a unique local descriptor encoding scheme [46] paired with a k-NN classifier achieved an 86.15% accuracy on *S. cerevisiae* data [45] while maintaining competitive performance on independent *E. coli* systems, demonstrating its utility as a supplementary predictive framework.

#### 4.1.4. Ensemble-Based PPIN Classification

To maximize predictive stability and eliminate single-model biases, contemporary investigators increasingly turn to hybrid ensemble classification frameworks. These systems frequently combine heterogeneous feature extraction strategies (including CT, AC, MAC, and localized descriptors) with Principal Component Analysis (PCA) for dimensionality reduction, then feed the condensed representations into ensemble Extreme Learning Machines to achieve a highly generalized 87% accuracy across *S. cerevisiae* and *E. coli* [50]. Alternatively, stacking strategies that consolidate SVM, RF, Multi-Layer Perceptron (MLP), and Naive Bayes (NB) models—utilizing an MLP as a meta-learner [52]—have been engineered to map intricate human–HCV interactomes by testing primary sequence, evolutionary conservation, post-translational modifications (PTMs), and native network properties, significantly outperforming single-classifier benchmarks [48].

### 4.2. Network Embedding-Based Approach

Let a biological network be represented as a graph G=(V,E), where V represents the set of nodes (proteins), and E signify the set of edges (interactions). The primary objective of network embedding is to learn a mapping function:

$$f:V\to R^{d},\mathrm{where}\;d<<|V|,$$

This optimization ensures that the structural topologies, local neighborhood contexts, and semantic relationships among nodes in the native graph are preserved within a low-dimensional, dense vector space. In this compressed embedding space, nodes that lie in close topological proximity within the original graph exhibit highly similar vector representations. By mapping high-dimensional discrete graphs into continuous vectors, network embedding enables the seamless application of downstream machine learning algorithms for tasks such as node classification, cluster detection, and link prediction.

#### 4.2.1. Matrix Factorization

Matrix factorization techniques decompose the interactome adjacency matrix into low-rank latent components to infer missing links. For instance, the symLMF framework [62] optimizes a logistic low-rank matrix factorization model specifically designed for link prediction within undirected protein–protein interaction networks (PPINs). To address host–pathogen systems, the VKBNMF model [66] implements a kernel Bayesian logistic matrix decomposition model equipped with automatic rank determination. This variational Bayesian formulation enables highly accurate predictions of human–virus protein interactions and exhibits robust cross-benchmark generalization. Furthermore, a sparse matrix completion framework utilizing Non-negative Matrix Tri-Factorization (NMTF) [49] has been engineered to predict novel interactions using positive-only data, successfully integrating heterogeneous biological data streams to outperform contemporary baselines on yeast networks.

#### 4.2.2. Random Walk

Random walk paradigms exploit localized and global stochastic routing across graph topologies to discover implicit node affinities. Recent implementations deploy continuous-time classical and quantum random-walk methodologies to systematically predict missing links in incomplete or noisy PPI networks [65]. Similarly, the Essentiality Ranking (EssRank) method [70] integrates interaction confidence scores, direct and indirect topological relationships, and random walk trajectories. By capturing multi-hop neighborhood features, this method accurately identifies essential proteins, significantly outperforming traditional network centrality measures and localized topology-based algorithms on yeast PPI benchmarks.

## 5. Deep Learning (DL) Approaches for PPI Prediction 

DL has attracted substantial attention across diverse scientific domains due to its ability to perform unsupervised feature learning, leading to prominent efforts to address PPI prediction. Computational methodologies in this domain are broadly bifurcated into geometry-aware architectures—which exploit graph neural networks (GNNs), graph attention networks (GATs), and graph autoencoders to preserve structural and relational configurations—and non-geometry-aware frameworks.

In the first section, we outline PPI computational models that use non-geometry-aware approaches, followed by a review of frameworks that leverage geometry-aware deep learning techniques for PPI prediction (Table 3, Figure 2).

### 5.1. Non-Geometry-Aware DL Approaches for PPI Prediction

Non-geometry-aware deep learning approaches treat proteins as independent entities, represented strictly by sequence or feature vectors, and seek to model the underlying interactive functions rather than explicit graph topologies. Within this paradigm, sequence-based feature extraction has been revolutionized by advanced language models (LMs) such as the LSTM-based SeqVec and the BERT-based ProtBert, which process a protein’s primary structure to generate dense, residue-level feature vectors that capture complex biophysical rules. When evaluated against standard benchmarks for human and *Saccharomyces cerevisiae*, these language models demonstrate outstanding predictive performance, outperforming contemporary legacy baselines.

Similarly, the Res2vec residue representation methodology [73] maps residue-to-residue transitions directly from raw sequences, providing highly optimized inputs for downstream deep learning classification frameworks without requiring any 3D structural parameters. The resulting end-to-end framework, DeepFE-PPI, achieved predictive accuracies of 94.78% on the *S. cerevisiae* dataset and 98.71% on human interactomes, while maintaining an average accuracy of 100% across five independent species datasets encompassing *H. sapiens*, *E. coli*, *M. musculus*, *H. pylori*, and *C. elegans* [73].

#### 5.1.1. Multi-Layer Perceptron (MLP)-Based Models

Historically, the earliest and most straightforward feedforward implementations utilized Multi-Layer Perceptrons (MLPs), which extract and concatenate static biological descriptors—including amino acid composition (AAC), dipeptide frequencies, physicochemical properties, domain interactions, Position-Specific Scoring Matrices (PSSMs), and Gene Ontology (GO)-based similarity metrics—to learn non-linear interaction boundaries. For instance, DeepAraPPI [81] integrates domain-level embeddings derived from Domain2vec with an MLP classifier to predict Arabidopsis PPIs with 91.3% accuracy.

To address the architectural inability of standard MLPs to capture long-range sequential contexts, the SDNN-PPI framework [84] integrates a self-attention mechanism with deep neural networks; by extracting global and local descriptors via AAC, conjoint triad (CT), and autocovariance (AC) formulations, the attention layers dynamically amplify salient features prior to classification. This network demonstrated strong generalization under 5-fold cross-validation, achieving accuracies of 95.48% on *S. cerevisiae* (core subset) and 98.94% on human intraspecific data, alongside interspecific accuracies of 93.15% on human–*Bacillus anthracis* and 88.33% on human–*Yersinia pestis* datasets.

To map host–pathogen boundaries, another variant employs a Multi-Layer Feed-Forward Network (MFFN) [72], a neural network that handles black-box classification by fusing macroscopic interactome features (node degree, betweenness centrality, and clustering coefficients) with microscopic sequence similarities and amino acid quadruplets, outperforming traditional SVM metrics on *B. anthracis* and Human Papillomavirus (HPV) data when all multi-scale feature combinations are utilized simultaneously during training.

#### 5.1.2. Convolutional Neural Network (CNN)-Based Models

Beyond feedforward networks, convolutional neural networks (CNNs) are widely deployed to extract invariant, localized sequence motifs and identify structural properties along the polypeptide chain. Xie et al. [76] used a CNN topology for PPI site prediction, utilizing residue binding propensities to enrich positive training instances and achieving a remarkable area under the curve (AUC) of 0.912.

This convolutional feature extraction strategy has been expanded to heterogeneous networks within the RPIFSE framework [74] for RNA–protein interaction prediction using sequence data, which extracts deep sequence features via a CNN, handles feature perturbation to generate multiple weighted datasets, and applies an Extreme Learning Machine (ELM) classifier within a weighted voting ensemble to yield 5-fold cross-validation accuracies of 91.87%, 89.74%, 97.76%, and 98.98% across the RPI369, RPI2241, RPI488, and RPI1807 datasets, respectively [85,86]. To directly capture the bidirectional influence between paired sequences, the PIPR model [87] employs a Siamese Residual Recurrent CNN architecture that combines local residual convolutional features with global contextual information in an end-to-end, sequence-only pipeline.

#### 5.1.3. Recurrent Neural Network (RNN)-Based Models

Complementing convolutional frameworks, recurrent neural networks (RNNs), including Long Short-Term Memory (LSTM) networks and Gated Recurrent Units (GRUs), are uniquely designed to resolve long-range sequential dependencies and contextual rules within amino acid sequences. For instance, a foundational LSTM pipeline developed in 2019 [75] mapped primary structures to numerical vectors using text-based protein signatures and a Prot2Vec (Protein2Vector) encoding strategy, outperforming alternative numerical mapping configurations in terms of ROC, log loss, and accuracy. This concept was advanced by Tsukiyama et al. using word2vec sequence encodings [88] inside an LSTM classifier, a strategy that directly inspired the development of LSTM-PHV [78].

Specifically engineered to map human–virus protein interactions strictly from raw sequences, LSTM-PHV handles highly skewed positive-to-negative sample distributions to deliver an AUC of 0.976 and 0.973 alongside accuracies of 0.984 and 0.985 on training and independent datasets, respectively, while outperforming existing baselines on entirely unknown or novel viral species. To address pandemic threats like COVID-19, researchers engineered a specialized mapping system [89] that normalizes and feeds distinct numerical descriptors—including Electron–Ion Interaction Potentials (EIIPs), Complex Prime Number Representations (CPNRs), and hydrophobicity scales—into a deep bidirectional recurrent neural network (DeepBiRNN), achieving an average accuracy of 97.76% in predicting physical interactions between SARS-CoV-2 viral proteins and human host receptors.

#### 5.1.4. Transfer-Based Models

The paradigm has been further advanced by transfer learning and transformer-based architectures, which leverage massive language models pre-trained on large sequence repositories. The ProBERT model [90], inspired by BERT, learns deep contextual embeddings from vast sequence data that transfer effectively to downstream prediction tasks, as demonstrated by the hybrid ProtBert-BiGRU-Attention model [91], which extracts amino acid features via ProtBERT, processes sequential context through a Bidirectional GRU, and applies an attention mechanism to isolate critical interactive features for highly accurate binary classification.

Similarly, the D-SCRIPT deep learning framework [79] tracks cross-species generalizability by mapping raw sequence strings into implicit structural contact representations to evaluate physical binding compatibility; this architecture was successfully utilized to screen the *Bos taurus* genome at a genome-wide scale to isolate functional metabolic and immune gene modules linked to rumen physiology. Furthermore, the Evolutionary Scale Modeling (ESM) framework [92] trains a deep contextual language model on 86 billion amino acids from 250 million structurally diverse, unlabeled protein sequences via self-supervised learning, automatically capturing multi-scale biological representations ranging from elemental physicochemical attributes to remote protein homologies to optimize downstream target prediction.

#### 5.1.5. Autoencoder-Based Models

In parallel, unsupervised architectures such as autoencoders are used to learn compact, low-dimensional latent spaces from raw features prior to interaction mapping. A notable configuration combines a Stacked Sparse Autoencoder (SSAE) with Legendre moment (LM) feature extraction, passing the compressed representations to a Probabilistic Classification Vector Machine (PCVM). The reported 5-fold cross-validation accuracies—98.58% for human, 97.71% for unbalanced human, 93.76% for *H. pylori*, and 96.55 for *S. cerevisiae*—are taken from Wang et al. [71], where this SSAE-LM-PCVM pipeline was compared with standalone SVM baselines.

Similarly, DeepPPI employs a deep feedforward network to extract clean, low-dimensional representations from common protein descriptors, securing a test accuracy of 92.50% [16]. Moving toward generative variations, variational autoencoders (VAEs) map protein features to probabilistic continuous latent representations to generate highly informative embeddings; this paradigm is exemplified by the Signed Variational Graph Autoencoder (S-VGAE) model [77], which synthesizes sequence-derived feature vectors with topological structural elements to yield robust latent representations tailored for multi-scale PPI prediction networks.

### 5.2. Geometry-Aware Deep Learning Approaches for PPI Prediction

Proteins do not function alone; they interact with each other to perform a specific biological process. Information on how different proteins coordinate to enable biological processes within the cell is primarily captured by the PPIN [93]. The theory of complex networks is fundamental to many fields, including molecular and population biology, engineering, physics, sociology, and computer science [94]. The potential applications of graphical network analysis include determining a protein’s or gene’s function, identifying potential drug targets, designing effective strategies for treating various diseases, and enabling early diagnosis of disorders [94]. Graph network-based analysis plays a significant role in understanding the essential topological characteristics of biological networks. An unweighted, undirected graph is a typical representation of a PPIN, where each node represents a protein and an edge between two nodes denotes that these proteins have been found to physically interact [95]. Representing a protein-protein network as a graphical network and studying its topological characteristics helps researchers understand and extract a large amount of information from the network.

**Figure 3:**
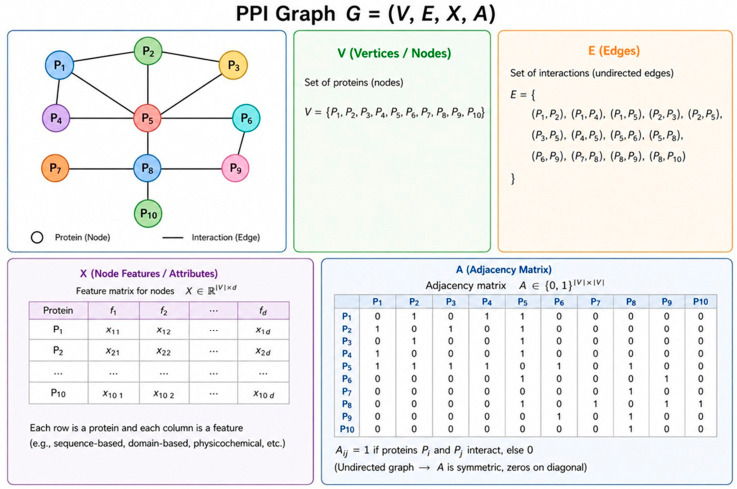
Mathematical and structural formulation of a protein–protein interaction (PPI) graph. The network is formally defined as a tuple *G =* (*V*, *E*, *X*, *A*) to prepare biological interaction data for graph-based machine learning pipelines. Top Left (Visual Graph Topology): Spatial visualization of the undirected PPI network where circles represent individual protein nodes (*P*_1_ … *P*_10_) and interconnecting lines represent physical or functional interactions (edges). Top Center (Node Set, *V*): Definition of the vertex set containing all ten unique proteins present in the system, bounding the dimensionality of the graph. Top Right (Edge Set, *E*): The set of unordered pairs representing undirected interactions between adjacent proteins (e.g., (*P*_1_, *P*_2_)), establishing the network’s structural connectivity. Bottom Left (Feature Matrix, *X*): A tabular matrix of dimensions |*V*| × *d*, mapping each protein row to a d-dimensional feature vector containing numerical attributes (e.g., amino acid sequence embeddings, structural domains, or physicochemical properties). Bottom Right (Adjacency Matrix, *A*): A square, binary matrix of size |*V*| × |*V*| where a value of 1 denotes a documented interaction between protein *Pi* and protein *Pj*, and 0 denotes no interaction. The matrix is symmetric with a zero diagonal due to the undirected nature of the graph.

Though we have provided a mathematical representation of the PPI network in the form of a graph, G=(V,E), from a deep learning perspective, we now generalize the graph representation of the PPI network as G=(V,E,X,A), where $V=\{v_{1},v_{2},...,v_{n}\}$ is the set of proteins (nodes) and a set of edges E⊆V×V represents the interaction between protein pairs (or pairs of nodes) [96]. Each node $v_{i}$ is associated with a d-dimensional feature vector $x_{i}$ (i.e., $x_{i}\in R^{d}$) that describes biological attributes such as sequence, structure, function, evolutionary profile, etc. The interactions are encoded by an adjacency matrix $A\subseteq R^{n\times n}$, where $A_{ij}=1$ if proteins i and j interact; otherwise $A_{ij}=0$. $X\in R^{n\times d}$ represents the node feature matrix, where *n* is the number of proteins and *d* is the number of associated features. This representation preserves both the topological and biological characteristics of proteins and enables graph neural network models to make better predictions and inferences. Figure 3 illustrates the general graphical architecture of protein–protein interaction that can be used as input to geometry-aware deep learning models for PPI prediction.

A graph neural network (GNN) learns low-dimensional node embeddings that preserve both the local and global network topology for efficient prediction of PPIs. A novel PPI model is proposed that uses graph structure and protein primary-structure information to generate protein representations for PPI prediction [97]. There are two main phases in this PPI prediction task: the representation phase and the prediction phase. Firstly, during the protein representation phases, protein sequences are one-hot encoded, and GCNs are used to capture graph-structural information. Following processing by the encoding and GCN modules, two matrices are obtained that contain, respectively, information about the protein sequence and the graph structure. The feature matrix and the adjacency matrix are input to the model. Then, the graph structure and protein sequence information are combined to get the final representation matrix of the proteins. To make PPI predictions, this combined matrix is fed into a fully connected deep neural network (DNN) to extract high-level features and make predictions. The proposed model has been validated using three datasets: human, yeast, and S. cerevisiae. The proposed method outperformed two existing sequence-based PPI models, DPPI [98] and DeepFE-PPI [73], demonstrating excellent predictive performance. Compared with the two existing PPI methods, the proposed method performed extremely well on the yeast dataset, outperforming DeepFE-PPI and DPPI. It was 2.54% and 5.78% more effective than DeepFE-PPI and DPPI. The proposed method also performed extremely well on the human dataset, in contrast to DeepFE-PPI and DPPI. The proposed method was 2.69% and 4.15% more effective than DeepFE-PPI and DPPI, respectively, on the human dataset. The proposed method also demonstrated competitive predictive performance on the *S. cerevisiae* dataset and outperformed the other two sequence-based PPI models with high accuracy. This proposed method uses a computationally simpler approach to protein representations and predicts with high accuracy compared with existing sequence-based complex PPI models.

Based on the underlying architectural design and learning mechanism, the geometry-aware deep learning approaches for PPI prediction can be subdivided into the following categories.

#### 5.2.1. Spatial-Based GNNs

Spatial GNNs learn embedding by sampling and aggregating information from local neighborhoods. This is scalable for large biological interaction networks [99,100]. Hamilton et al. [99] proposed GraphSAGE, a general inductive framework that leverages node features (e.g., text attributes) to efficiently generate node embeddings for previously unseen data. They classify unseen nodes in evolving information graphs using citation and Reddit post data. The single embedding $\left(h_{v}^{k}\right)$ of node *v* in layer *k* in GraphSAGE by aggregating the feature information from a sample neighborhood is done by the following rule:

$$h_{v}^{\left(k\right)}=\sigma (W^{\left(k\right)}CONCAT(h_{v}^{\left(k-1\right)},AGG(h_{u}^{\left(k-1\right)}|u\in N_{s}(v))),$$

where $\left(h_{v}^{k}\right)$ is the representation of node *v* at layer *k*, *N(v)* denotes the set of neighboring nodes of *v*, *AGG* represents the aggregation function, *W* is the learnable weight matrix, and sigma (*σ*) is the activation function. Additionally, GNNs based on graph structures and message passing (MPNN) adeptly capture local patterns and global relationships in protein structures [101]. Zhang and Chen [102] presented a new theory for learning link prediction heuristics, justifying learning from local subgraphs rather than entire networks, and proposed SEAL, a novel link prediction framework based on GNNs. The authors in [103] introduced DL-PPI, a novel deep learning framework for sequence-based PPI prediction. It improves feature extraction from individual protein sequences and captures inter-protein relationships using a novel Feature Relationship Network (FRN) based on graph neural networks, thereby improving PPI prediction accuracy.

#### 5.2.2. Spectral-Based GNNs

Jha et al. [104] employ graph neural networks (GNNs), such as the graph convolutional network (GCN), to predict protein interactions using proteins’ structural and sequence characteristics. The PDB files are used to build protein graphs, which contain 3D coordinates of atoms. The amino acid network, also known as the residue contact network, is a protein graph in which each amino acid residue is a node, and the connections between them are edges. If amino acids have a pair of atoms (one from each amino acid) within the threshold distance, they are said to be connected. Fout et al. [105] proposed a spatial graph convolution model that combines learned features across protein pairs to classify amino acid residue pairs as part of an interface. The architecture proposed here predicts protein-protein interfaces using a graph representation of the underlying protein structure. Here, the GCN learns node embedding by aggregating the neighborhood information layer-wise by using the following rule:

$$H^{\left(k+1\right)}=\sigma ({\tilde{D}}^{-1/2}\tilde{A}{\tilde{D}}^{-1/2}H^{\left(k\right)}W^{\left(k\right)}),$$

where $H^{0}=X$, $H^{\left(k\right)}\in R^{n\times d}$ is the node embedding matrix at layer *k*, *W^(k)^* is a trainable weight matrix, *D* is the degree matrix, and *σ* is an activation function.

#### 5.2.3. Attention-Based GNNs

An attention-based graph neural network model assigns different weights to neighbors based on their attention scores. Baranwal et al. [80] proposed a novel multi-layer mutual graph attention network (GAT)-based architecture, termed Struct2Graph, for the task of PPI prediction. The model takes as input coarse-grained structural representations of a given protein pair and produces the probability of interaction between the two proteins. Struct2Graph employs two graph convolutional networks (GCNs) with shared weights, along with a mutual attention mechanism, to capture and learn important geometric features that characterize the interaction patterns between the query protein pair. Lai and Xu [106] developed GAT-GO, which uses predicted structural information and ESM-1b embeddings for protein function prediction; it is therefore best treated as an adjacent structure-aware graph-learning example rather than a direct pairwise PPI predictor. To more effectively extract protein information for predicting multi-type PPIs, two attention network frameworks were integrated to construct a learning network termed AFTGAN [82]. This study assigns different weights to relationships among protein nodes, more effectively capturing protein relationships through the attention mechanism. The embedding of node or protein *i* at layer *k* is computed as a weighted combination of neighbor features: $h_{i}^{\left(k+1\right)}=\sigma (\sum_{j\in N(i)}\alpha_{ij}Wh_{j}^{\left(k\right)})$. To improve stability, multiple attention heads are used for averaging in the final layer using the following formula for protein *i* at layer (*k +* 1):

$$h_{i}^{\left(k+1\right)}=\sigma \left(\frac{1}{M}\sum_{i=1}^{M}\sum_{j\in N(i)}\alpha_{ij}^{M}W^{m}h_{j}^{\left(k\right)}!\right)$$

#### 5.2.4. Graph Autoencoders

Yang et al. [77] considered the PPI network as an undirected graph and proposed a model based on a signed variational graph autoencoder (S-VGAE) and a conjoint triad (CT) that effectively leverages the naturally incorporated protein sequence information and graph structure as features. The overall framework is composed of three parts. Firstly, the raw protein sequences were encoded using CT methods; secondly, vector embeddings for each protein were extracted from both sequence information and graph structure using the essential S-VGAE model. A simple three-layer feed-forward neural network is used for the classification of protein–protein interactions, and to train it, the S-VGAE model embeddings were used as input. The proposed model achieved high accuracy on the Human Protein Reference Database (HPRD) dataset and obtained the best results on the Caenorhabditis elegans (*C. elegans*), Database of Interacting Proteins (DIP), and Human, Drosophila, and Escherichia coli (*E. coli*) datasets. The model’s prediction capability is also validated against existing PPI methods, and it outperforms all existing models with excellent accuracy. Variational autoencoder (VGAE) extends the graph autoencoder by learning probabilistic latent embeddings of proteins in a PPI network. It models uncertainty in protein representations, which improves predictions of unknown interactions. Mathematically, instead of learning a deterministic embedding, VGAE learns the following distribution:

$$q(Z|X,A)=\prod_{i=1}^{n}Ν(z_{i}|\mu_{i},diag(\sigma_{i}^{2})),$$

where *z_i_* is the latent embedding of protein *i*, *μ_i_* is the mean vector, and *σ_i_* is the standard deviation of the Gaussian normal distribution *N*.

#### 5.2.5. Dynamic/Temporal GNNs

Dynamic or temporal graph neural networks have been applied to PPI prediction to capture evolving interaction patterns across biological conditions. Chen et al. [83] proposed DCMF-PPI, which models temporal variations in protein structure and interaction networks using dynamic adjacency matrices and geometry-aware learning techniques. DCMF-PPI is a hybrid framework that combines dynamic modeling, multi-scale feature extraction, and probabilistic graph representation learning. It includes a PortT5-GAT module, where the PortT5 protein language model extracts residue-level features, incorporates temporal dependencies, and uses graph attention networks to capture context-aware structural variations in protein interactions. In temporal GNNs, protein representations evolve over time by integrating both structural and temporal dependencies. The general temporal update rule is [83]: $h_{i}^{\left(t+1\right)}=f(h_{i}^{\left(t\right)},G_{t})$, where $h_{i}^{\left(t\right)}$ denotes embedding of protein *i* at time step *t*, *f(.)* represents temporal graph learning function combining graph structure historical information, and $G_{t}=(V_{t},E_{t},X_{t})$ denotes the graph at time step *t* where *V_t_* is the set of proteins, *E_t_* represents PPI, and *X_t_* denotes the node feature matrix at time step *t*. The other dynamic GNN models incorporate recurrent neural network (RNN) to capture temporal dependencies using the following formula [107,108]:

$$h_{i}^{\left(t+1\right)}=GRU(h_{i}^{\left(t\right)},GNN(A_{t},X_{t}{)}_{i}),$$

where *A_t_* is the adjacency matrix at time step *t*, and $GNN(A_{t},X_{t})$ computes the structural embedding of nodes from a graph snapshot.

#### 5.2.6. Heterogeneous GNNs

Heterogeneous graph neural networks (Het-GNNs) are designed to model multi-type biological entities and relationships that naturally arise in bioinformatics data. In PPI prediction, heterogeneous graphs enable the integration of diverse biological information, such as proteins, genes, drugs, diseases, pathways, and functional annotations, within a unified framework. This kind of integration improves predictive performance by capturing complementary biological knowledge from multiple sources. Heterogeneous PPI networks comprise multiple node types and interaction types, providing a richer representation than homogeneous graphs. A heterogeneous biological interaction network represented as $G=(V,E,X,\Gamma_{v},\Gamma_{e})$, where *V* is the set of proteins, *E* is the set of interactions, *X* is the node feature matrix, $\Gamma_{v}$ is the set of different node types, and $\Gamma_{e}$ is the set of different edge types. In heterogeneous GNNs [108], neighbors are grouped based on relation type. For node *i*, neighbor under relation *r* is defined as

$N_{r}(i)=\{j|(i,j,r)\in E\}$, where (i,j,r)∈E indicates that there exists an edge between nodes *i* and *j* with relation type *r*. The message aggregation for each relation is defined as

$$m_{i}^{\left(k,r\right)}=\sum_{j\in N_{r}(i)}\frac{1}{C_{i,r}}W_{r}^{\left(k\right)}h_{j}^{\left(k-1\right)},$$

where $W_{r}^{\left(k\right)}$ is the relation-specific weight matrix, $C_{i,r}$ is the normalized constant, and $h_{j}^{\left(k-1\right)}$ is the embedding of the neighbor node. The compact mathematical formulation for heterogeneous GNN is [100,108]

$$H^{\left(k+1\right)}=\sigma \left(\sum_{r\in \Gamma_{e}}D_{r}^{-1}A_{r}H^{\left(k\right)}W_{r}^{\left(k\right)}\right)$$

where *A_r_* is the adjacency matrix for relation type *r*, *D_r_* is the degree matrix for relation *r*, $W_{r}^{\left(k\right)}$ is a learnable parameter matrix, and *σ* is a nonlinear activation function. Zitnik et al. [100] developed Decagon, a method for predicting side effects of drug pairs by constructing a two-layer multimodal graph comprising protein–protein, drug–protein, and drug–drug interactions (side effects). Each drug–drug interaction is represented as a distinct edge type corresponding to a specific side effect. A multi-relational GCN was then developed to predict both drug–drug interactions and their associated side effect types. Exploratory analysis shows that co-prescribed drug pairs often share common target proteins, indicating that drug–protein interaction information is valuable for modeling drug combinations.

#### 5.2.7. Structure- or Residue-Aware GNNs

Structure-aware or residue-aware GNNs use 3D structural information of proteins to improve PPI predictions. Instead of representing proteins only at the sequence level, these approaches construct a graph in which nodes correspond to amino acid residues and edges represent spatial proximity or biochemical relationships derived from protein tertiary structures. Such structural representations enable the capture of geometric and physicochemical patterns that determine binding affinity and interaction specificity. Here, the protein structure graph can be represented as $G=(V_{r},\;E_{r},\;X_{r})$, where *V_r_* is the set of amino acid residues, *E_r_* is the set of structural connections between residues, and *X_r_* is the residue feature matrix. The structure-aware message passing incorporates the spatial relationships in the embedding space, which is defined as

$$h_{i}^{\left(k+1\right)}=\sigma \left(\sum_{j\in N(i)}W^{\left(k\right)}h_{j}^{\left(k\right)}\right)$$

$$h_{i}^{\left(k+1\right)}=\sigma \left(\sum_{j\in N(i)}\phi \left(h_{i}^{\left(k\right)},h_{j}^{\left(k\right)},e_{ij}\right)\right)$$

where *ϕ*(.) is the geometric message function, and *e_ij_* is the spatial relation between residues. Jing et al. in [109] introduce Geometric Vector Perceptrons (GVPs), which extend standard dense layers to operate on collections of Euclidean vectors and serve as a drop-in replacement for Multi-Layer Perceptrons (MLPs) in GNN aggregation and feed-forward layers. GVPs process both scalar and geometric features that transform consistently under spatial rotations, enabling effective learning from protein structural data. The authors demonstrate the approach on protein structure-related tasks, including computational protein design (CPD), which aims to predict an amino acid sequence that folds into a given structure, and model quality assessment. Another work on end-to-end learning for 3D protein structure-based interface prediction is presented by Townshend et al. [110], who introduced DIPS, a dataset for interface prediction that is two orders of magnitude larger than previously available datasets. To address the limitations of hand-crafted features in handling dataset bias, they propose SASNet, the first end-to-end deep learning framework for protein interface prediction.

## 6. Other Methods

### 6.1. Bayesian and Probabilistic Models

Bayesian and probabilistic models provide a principled framework for modeling uncertainty and integrating heterogeneous biological data in PPI prediction. Unlike deterministic approaches, probabilistic methods explicitly represent uncertainty in both data and model parameters, making them particularly suitable for biological datasets that are often noisy, incomplete, and high-dimensional (Table 4, Figure 2).

In the Bayesian framework, the probability of interaction between a pair of proteins is inferred by combining prior knowledge with observed data using Bayes’ theorem. Formally, given a protein pair (*i*, *j*) and observed features *x_**ij**_* derived from sequence, structural, network, or functional information, the posterior probability of interaction can be expressed as

$$P(y_{ij}=1|x_{ij})=\frac{P(x_{ij}|y_{ij}=1)P(y_{ij}=1)}{P(x_{ij})},$$

where *y_ij_* denotes the interaction label. This formulation enables the integration of multiple evidence sources while accounting for their relative contributions and uncertainties.

Several studies illustrate how probabilistic and network-based evidence can support PPI inference, although not all are direct pairwise PPI classifiers. Liu and Rajapakse [111] proposed a framework for gene-regulatory-network inference that fuses gene-expression data with transitive PPIN information; it should therefore be interpreted as a GRN/PPIN evidence-fusion method rather than a standard PPI-pair predictor. Wang et al. [112] introduced ActivePPI, a Markov-random-field-based framework for quantifying condition-specific PPIN activity from proteomics abundance data, rather than an active-learning binary PPI classifier. Earlier work by Morshed et al. [113] employed Gaussian process-based probabilistic models to integrate multiple genomic and proteomic data sources, demonstrating improved robustness and generalization.

### 6.2. Temporal PPINs

Temporal PPINs aim to capture these time-varying interaction patterns, providing a more realistic and informative representation of cellular processes. A temporal PPIN can be modeled as a sequence of time-indexed graphs ${\left\{G_{t}=\left(V_{t},E_{t}\right)\right\}}_{t=1}^{T}$, where *V_t_* and *E_t_* denote the sets of proteins and interactions at time *t*, respectively. Alternatively, continuous-time formulations represent interactions as timestamped events, enabling fine-grained modeling of interaction dynamics. Such representations enable the analysis of network evolution, the identification of transient interactions, and the detection of temporal functional modules.

Recent advances have focused on integrating heterogeneous temporal data sources and developing sophisticated models for dynamic PPI prediction. For instance, Li et al. [114] proposed a temporal modeling framework that captures dynamic interaction patterns using time-aware representations. Chen et al. [83] introduced a dynamic collaborative matrix factorization approach to model temporal dependencies and latent interaction structures.

In addition, the construction and reconstruction of temporal PPINs from noisy and incomplete data have been extensively studied. Li et al. [115] proposed methods for constructing temporal networks from time-series biological data, while He et al. [116] focused on reconstructing dynamic interaction networks using advanced inference techniques. These approaches highlight the importance of incorporating temporal information to better understand the evolution of biological systems. Table 4 summarizes Bayesian, probabilistic, and temporal PPIN-related methods discussed above.

Direct numerical comparisons across studies should be interpreted with caution because reported performance depends strongly on benchmark construction, negative-sampling strategy, class balance, split protocol, and evaluation metric. Accordingly, the comparative tables in this review are intended as structured summaries of the literature rather than strict cross-study rankings.

## 7. Discussion and Future Directions

The synthesis of computational frameworks for human–virus PPI prediction shows a shift from descriptive, low-throughput experimental mapping toward predictive, multi-scale in silico modeling. Traditional feature-based ML approaches offer computational efficiency and useful baseline performance when mapping interactions from sequence-derived physicochemical descriptors, but they remain limited by manual feature engineering and by their restricted capacity to capture long-range contextual or spatial dependencies. In contrast, DL models can learn hierarchical representations from sequences, structures, and interaction networks. Within the sequential domain, large-scale pre-trained protein LMs capture evolutionary patterns and remote homologies directly from raw sequences, whereas geometry-aware GNNs preserve interactome topology and spatial configurations.

However, evaluation of these frameworks highlights unresolved bottlenecks that limit biological and translational reliability. Public repositories remain noisy, biased, and sparse in experimentally validated true-negative interactions, forcing many models to rely on synthetic negative sampling, which can inflate reported accuracy. Many high-performing DL models also function as mathematical “black boxes,” offering limited biochemical or residue-level interpretation of why a given interaction is predicted, thereby restricting their use in targeted drug design or vaccine-target prioritization. Finally, many frameworks treat the interactome as a static binary graph, whereas viral biology is context-dependent: mutation, post-translational modification, cellular localization, tissue tropism, and host immune state can all alter binding affinity.

A further limitation is that the review is centered on human–virus systems with substantial emphasis on coronavirus-related studies. This focus is justified by the scale of SARS-CoV-2 research, but models trained or benchmarked in coronavirus settings may not transfer uniformly to viral families with different replication strategies, genome organization, tissue tropism, latency patterns, integration mechanisms, or host-entry pathways. For example, enveloped RNA viruses, retroviruses, herpesviruses, papillomaviruses, and large DNA viruses can interact with host proteins through distinct structural motifs and immune evasion mechanisms. Future studies should therefore evaluate generalization using virus-wise, family-wise, and external test splits; report performance separately for unseen viral proteins or families; and use transfer learning, domain adaptation, calibrated uncertainty estimates, and multimodal sequence-structure-network representations to improve robustness beyond coronavirus-centered benchmarks.

In summary, ML and DL methods have substantially expanded the computational toolkit for human–virus PPI prediction, but robust progress depends on more than architectural novelty. Better-curated virus–host interaction resources, transparent negative-sampling protocols, protein- and virus-wise evaluation splits, uncertainty reporting, and interpretable multimodal models are needed to distinguish biologically reliable predictions from benchmark-specific performance gains. Future frameworks that integrate sequence language models, structural modeling, graph learning, and experimentally validated external testing are likely to provide the most useful route toward generalizable prediction of host–virus interactions across diverse viral families.

## Figures and Tables

**Table 1:** Curated biological databases and resources commonly utilized in computational human–virus protein–protein interaction prediction.

Resource Category	Database/Resource	Resource Type	Primary Application in Human–Virus PPI Prediction	Data Modality	Organism Scope	Curation Status	Representative Use in Computational Frameworks	Official URL
**Protein Sequence Resources**	UniProtKB/Swiss-Prot	Curated protein knowledge base	Protein annotation, functional characterization, sequence retrieval	Protein sequence, functional annotation	Multi-species	Expert-curated	Sequence embedding, feature engineering, transformer-based protein modeling	https://www.uniprot.org/
	TrEMBL	Computationally annotated protein repository	Large-scale sequence acquisition for deep learning pipelines	Protein sequence	Multi-species	Automatically annotated	Pretraining and large-scale sequence representation learning	https://www.uniprot.org/
	PIR	Protein information repository	Protein family and functional classification	Protein sequence and annotation	Multi-species	Curated	Evolutionary feature extraction and comparative sequence analysis	https://proteininformationresource.org/
**Structural Biology Databases**	PDB	Structural repository	Three-dimensional structural modeling of interacting proteins	3D protein structures	Multi-species	Curated experimental structures	Structure-aware and geometry-aware PPI prediction	https://www.rcsb.org/
	SCOP	Structural classification database	Protein fold and domain classification	Structural hierarchy	Multi-species	Curated	Structural similarity learning and fold-aware modeling	http://scop.mrc-lmb.cam.ac.uk/
	CATH	Protein domain architecture resource	Hierarchical structural classification	Structural topology and domains	Multi-species	Curated	Domain-aware graph representation learning	http://www.cathdb.info/
**Gene Ontology and Functional Annotation Resources**	Gene Ontology (GO)	Controlled biological ontology	Functional feature extraction and semantic similarity analysis	Biological process, molecular function, cellular component annotations	Multi-species	Curated	GO-based feature engineering and functional interaction modeling	http://geneontology.org/
	QuickGO	GO annotation browser	Rapid GO annotation retrieval	Functional annotations	Multi-species	Curated	Functional enrichment and annotation mapping	https://www.ebi.ac.uk/QuickGO/
	DAVID	Functional annotation platform	Functional clustering and pathway enrichment	Gene and protein annotations	Multi-species	Curated	Biological interpretation and pathway analysis	https://david.ncifcrf.gov/
**Protein–Protein Interaction Databases**	STRING	Integrated interaction network database	Known and predicted interaction retrieval	Physical and functional PPIs	Multi-species	Integrated curated + predicted	Network construction and graph-based learning	https://string-db.org/
	BioGRID	Interaction repository	Experimentally validated molecular interactions	Physical and genetic interactions	Multi-species	Curated	Benchmark dataset generation and validation	https://thebiogrid.org/
	IntAct	Molecular interaction database	Experimentally curated molecular interactions	Protein and molecular interactions	Multi-species	Expert-curated	Gold-standard interaction benchmarking	https://www.ebi.ac.uk/intact/home
	DIP	Database of interacting proteins	Experimentally verified PPIs	Protein interactions	Multi-species	Curated	Classical benchmark dataset construction	https://dip.doe-mbi.ucla.edu/dip/Main.cgi

Abbreviations: CATH, Class, Architecture, Topology, Homologous Superfamily; CC, Cellular Component (Gene Ontology Domain); DAVID, Database for Annotation, Visualization and Integrated Discovery; DIP, Database of Interacting Proteins; GO, Gene Ontology; HOS, Higher-Order Structure; HPRD, Human Protein Reference Database; MF, Molecular Function (Gene Ontology Domain); MINT, Molecular Interaction Search Tool; MIPS, Munich Information Center for Protein Sequences; PDB, Protein Data Bank; PIR, Protein Information Resource; PPIN, Protein–Protein Interaction Network; SCOP, Structural Classification of Proteins; STRING, Search Tool for the Retrieval of Interacting Genes/Proteins; TrEMBL, Translated EMBL Nucleotide Sequence Data Bank; UniProt, Universal Protein Resource; URL, Uniform Resource Locator.

**Table 2:** Representative machine learning-based frameworks for protein–protein interaction prediction and human–virus PPI modeling.

Study	Interaction Type	Organism/System	Model Framework	Feature Representation	Negative Sampling	Split Protocol	Validation Strategy	Primary Metrics	Reported Performance	Methodological Remarks
Shen et al. (2007) [14]	General PPIN	General PPIN	SVM	Conjoint triad encoding	Random negatives	Random pair split	5-fold CV	Accuracy	83.90%	Early sequence-based PPI prediction framework
Guo et al. (2008) [45]	General PPIN	*S. cerevisiae*	SVM	Autocovariance descriptor	Random negatives	Random pair split	Cross-validation	Accuracy	87.36%	Introduced AC-based sequence representation
Yang et al. (2010) [46]	General PPIN	*S. cerevisiae*	SVM/KNN	Local descriptor	Random negatives	Random pair split	Cross-validation	Accuracy	86.15%	Local descriptor + KNN/SVM on *S. cerevisiae* dataset
Dyer et al. (2011) [47]	Human–virus PPI	Human–virus interactions	Linear SVM	Domain profile + k-mer	Not reported	Not reported	Cross-validation	Recall	65.50%	Integrated host and viral descriptors
Cui et al. (2012) [48]	Human–virus PPI	Human–virus PPIs	RBF-SVM	Conjoint triad encoding	Not reported	Not reported	Cross-validation	Accuracy	87.50%	Applied CT encoding to host–virus interactions
Wang et al. (2012) [49]	General PPIN	Not reported	NMTF	Sparse matrix completion	Not reported	Not reported	Not reported	Precision	38.19%	Non-negative Matrix Tri-Factorization (NMTF)-based sparse matrix completion method
You et al. (2013) [50]	General PPIN	General PPIN	Extreme Learning Machine	Multiple sequence descriptors	Random negatives	Random pair split	Cross-validation	Accuracy	87.50%	Ensemble descriptor integration
Dey et al. (2020) [51]	Human–virus PPI	SARS-CoV-2–human	Sequence-based ML classifiers	AAC, pseudo AAC, conjoint triads	Not reported	Not reported	Not reported	Accuracy	72.33%	Sequence-based viral-host interaction prediction
Emamjomeh et al. (2014) [52]	Human–virus PPI	Human–HCV	Ensemble (SVM, RF, MLP, NB)	Not reported	Not reported	Not reported	Not reported	Accuracy	83.00%	Ensemble stacking of SVM, RF, MLP, and NB
Huang et al. (2015) [53]	General PPIN	Not reported	WSRC	Not reported	Not reported	Not reported	Not reported	Accuracy	96.28%	WSRC classifier
You, Chan & Hu (2015) [54]	General PPIN	Not reported	Random Forest	Multi-scale local features	Not reported	Not reported	Not reported	Accuracy	94.72%	Used a multi-scale local feature representation scheme and the Random Forest
Xu et al. (2019) [55]	Interaction ranking/ essential-protein detection	General PPIN	EssRank	Random-walk ranking	Not applicable	Not applicable	Confidence evaluation	PR-AUC	44.8%	Related network-ranking method, not a direct PPI classifier
Wang et al. (2017) [56]	General PPIN	Not reported	Not reported	Legendre moments (PSSM)	Not reported	Not reported	Not reported	Accuracy	96.28%	Used Legendre moments descriptor to extract discriminatory information embedded in PSSM
Wang et al. (2017) [57]	General PPIN	Not reported	PCVM	Zernike moments	Not reported	Not reported	Not reported	Accuracy	94.48%	Used a probabilistic classification vector machine model combined with a Zernike moments descriptor
Alguwaizani et al. (2018) [58]	Human–virus PPI	Virus–host proteins	SVM	AAC + repeat patterns	Random negatives	Independent test set	Independent testing	ROC-AUC	94.00%	Improved sequence discrimination
Song et al. (2018) [59]	General PPIN	Not reported	Ensemble classifier	Random projection	Not reported	Not reported	Not reported	Accuracy	95.64%	An ensemble classifier with random projection using sequence and evolutionary information
Lian et al. (2019) [60]	Human–pathogen PPI	Human–*Yersinia pestis*	RF + noisy-OR ensemble	Ensemble feature integration	Not reported	Not reported	Cross-validation	ROC-AUC	95.00%	Pathogen-specific interaction prediction
Yang et al. (2020) [61]	General PPIN	General PPIN	Random Forest	Doc2Vec embeddings	Not reported	Random pair split	Cross-validation	ROC-AUC	87.00%	Embedding-based sequence learning
Pei et al. (2021) [62]	General PPIN	Not reported	SymNMF	Matrix factorization	Not reported	Not reported	Not reported	ROC-AUC	94.00%	SymNMF matrix factorization model for PPIN link prediction
Debnath et al. (2022) [63]	General PPIN	Not reported	Linear SVM	Primary sequence info	Not reported	Not reported	Not reported	Accuracy	63.00%	Linear SVM using primary protein sequence information
Pan et al. (2022) [64]	Human PPIN	Human PPIN	Rotation Forest	DHT descriptor	Random negatives	Independent test set	Independent testing	ROC-AUC	98.00%	Robust handcrafted descriptor engineering
Goldsmith et al. (2023) [65]	General PPIN	Not reported	Random walk	Not reported	Not reported	Not reported	Not reported	ROC-AUC	92.00%	Continuous-time classical and quantum random walk methods
Ma et al. (2024) [66]	Human–virus PPI	Not reported	VKBNMF	Matrix decomposition	Not reported	Not reported	Not reported	ROC-AUC	89.00%	Kernel Bayesian nonlinear matrix factorization (VKBNMF)-based Bayesian logistic matrix decomposition model (Avg F1 = 85%)

Abbreviations: AAC, Amino Acid Composition; AC, Autocovariance; AUC, Area Under the Receiver Operating Characteristic Curve; Avg, Average; BiGRU, Bidirectional Gated Recurrent Unit; CKSAAP, Composition of K-Spaced Amino Acid Pairs; CNN, Convolutional Neural Network; CT, Conjoint Triad; DHT, Discrete Hartley Transform; Doc2Vec, Document to Vector; ESM, Evolutionary Scale Modeling; EssRank, Essentiality Ranking; F1, F1-Score (Harmonic Mean of Precision and Recall); HPV, Human Papillomavirus; HCV, Hepatitis C Virus; HIV, Human Immunodeficiency Virus; KNN, k-Nearest Neighbor; LSTM, Long Short-Term Memory; ML, Machine Learning; MLP, Multi-Layer Perceptron; NA, Not Available/Not Reported in the Original Study; NB, Naive Bayes; NMTF, Non-negative Matrix Tri-Factorization; PCA, Principal Component Analysis; PCVM, Probabilistic Classification Vector Machine; PPI, Protein–Protein Interaction; PseTC, Pseudo-Transition Composition; PSSM, Position-Specific Scoring Matrix; PTM, Post-Translational Modification; RBF, Radial Basis Function (Kernel); RF, Random Forest; RCNN, Recurrent Convolutional Neural Network; RoF, Rotation Forest; RNN, Recurrent Neural Network; SSAE, Stacked Sparse Autoencoder; SVM, Support Vector Machine; symLMF, Symmetric Logistic Matrix Factorization; VKBNMF, Variational Kernel Bayesian Non-negative Matrix Factorization; Word2Vec, Word to Vector; WSRC, Weighted Sparse Representation-Based Classification.

**Table 3:** Representative deep-learning frameworks for protein–protein interaction prediction.

Study	Task Category	Architecture	Model Family	Biological Context	Validation Strategy	Primary Metrics	Reported Performance	Methodological Remarks
Du et al. (2017) [16]	General PPIN	DeepPPI	Deep neural network	General PPIN	Cross-validation	ROC-AUC, MCC	AUC: 97.4%; MCC: 85.1%	End-to-end deep representation learning
Wang et al. (2018) [71]	General PPIN	SSAE + LM + PCVM	Deep feature learning	General PPIN	5-fold CV	Accuracy	Human: 98.58%; unbalanced human: 97.71%; *H. pylori*: 93.76%; *S. cerevisiae*: 96.55%	SSAE + LM + PCVM
Ahmed et al. (2018) [72]	General PPIN	MFFN	Feedforward neural network	General PPIN	Cross-validation	ROC-AUC	93.0%	Multi-descriptor neural architecture
Yao et al. (2019) [73]	General PPIN	DeepFE-PPI	Non-geometry-aware DL	Human + *S. cerevisiae*	5-fold CV	Accuracy, MCC	Accuracy: 98.7%; MCC: 97.4%	Sequence embedding framework
Wang et al. (2019) [74]	Related task: RNA-protein interaction	RPIFSE	Feature-selection ensemble	RPI datasets	Cross-validation	Accuracy	89.7–99.0%	Methodologically related RPI framework; not a direct pairwise PPI benchmark
Alakus et al. (2019) [75]	General PPIN	LSTM-Prot2Vec	Recurrent neural network	General PPIN	Cross-validation	Accuracy	92.0%	Context-aware embedding model
Xie et al. (2020) [76]	Related task: PPI-site prediction	CNN-based predictor	Convolutional neural network	Binding-site datasets	Cross-validation	ROC-AUC	91.2%	Site-level interaction prediction; not a pairwise PPI benchmark
Yang et al. (2020) [77]	Structure-aware PPIN	S-VGAE	Variational graph autoencoder	Signed interaction networks	Cross-validation	ROC-AUC, MCC	AUC: 99.9%; MCC: 98.3%	Geometry-aware graph learning
Tsukiyama et al. (2021) [78]	Human–virus PPI	LSTM-PHV	Sequence DL	Host–virus datasets	Independent testing	ROC-AUC	97.3%	Host–virus interaction prediction
Sledzieski et al. (2021) [79]	Cross-species PPI	D-SCRIPT	Transfer learning	Cross-species datasets	External validation	Recall	96.0%	Transferable cross-species prediction
Baranwal et al. (2022) [80]	Structure-aware PPIN	Struct2Graph	Graph Attention Network	Structural protein datasets	Independent testing	ROC-AUC, MCC	AUC: 99.9%; MCC: 98.8%	Structure-aware graph interaction modeling
Zheng et al. (2023) [81]	Plant PPIN	DeepAraPPI	Deep learning framework	Arabidopsis datasets	Task-specific validation	ROC-AUC	82.5–96.5%	Plant-specific interaction prediction
Kang et al. (2023) [82]	Human PPIN	AFTGAN	Transformer + GAT	SHS27k dataset	Random partition validation	Micro-F1	86.7%	Attention-based graph transformer
Chen et al. (2025) [83]	Dynamic PPIN	DCMF-PPI	Collaborative matrix factorization	SHS27k dataset	Random partition validation	ROC-AUC, MCC	AUC: 99.9%; MCC: 98.4%	Dynamic interaction modeling

Abbreviations: AFTGAN, Attention-based Feature Transformer Graph Attention Network; AUC, Area Under the Receiver Operating Characteristic Curve; CNN, Convolutional Neural Network; DCMF-PPI, Deep Collaborative Matrix Factorization for Protein–Protein Interaction; D-SCRIPT, Deep Sequence-Contact Representations Identifying Partner Targets; DeepAraPPI, Deep Learning for Arabidopsis Protein–Protein Interaction; DeepFE-PPI, Deep Feature Embedding for Protein–Protein Interaction Prediction; GRU, Gated Recurrent Unit; LM, Language Model; LSTM, Long Short-Term Memory; MCC, Matthews Correlation Coefficient; MFFN, Multi-Layer Feed-Forward Network; NA, Not Available/Not Reported in the Original Study; PCVM, Probabilistic Classification Vector Machine; PIPR, Protein–Protein Interaction Prediction Based on Siamese Residual RCNN; RPI, RNA–Protein Interaction; RPIFSE, RNA–Protein Interaction Feature Selection Ensemble; S-VGAE, Signed Variational Graph Autoencoder; SSAE, Stacked Sparse Autoencoder; VM, Vector Machine.

**Table 4:** Representative Bayesian, probabilistic, and temporal protein–protein interaction network frameworks.

Study	Framework Category	Biological Context	Temporal Modeling	Split Protocol	Validation Strategy	Primary Metrics	Standardized Performance	Methodological Contribution	Key Limitations
Chen et al. (2025) [83]	Dynamic matrix factorization	SHS27k dataset	Yes	Random partition	Random partition validation	F1-score	F1-score: 89.0%	Dynamic collaborative interaction modeling	Random partition may inflate apparent performance; external virus-wise or protein-wise validation is recommended
Liu et al. (2019) [111]	Related probabilistic GRN/PPIN feature fusion	Gene regulatory network inference with PPIN input	No	Random split	Cross-validation in the GRN setting	AUROC, AUPR	AUROC: 69.0%; AUPR: 50.0%	Fuses gene expression with transitive PPIN evidence	Not a direct pairwise PPI classifier
Wang et al. (2023) [112]	Network-activity modeling with Markov random fields	Condition-specific PPIN activity	Partial	Unknown	Not reported	Activity significance	Score range: 76–394	Quantifies PPIN activity using proteomics abundance data	Conventional PPI-classification metrics absent; interpret as network-activity modeling
Morshed et al. (2012) [113]	Bayesian co-learning	IRMA OFF dataset	No	Random split	Cross-validation	Precision, recall	Precision: 50.0%; recall: 75.0%	Probabilistic Gaussian process integration	Limited scalability
Li et al. (2024) [114]	Temporal representation learning	Dynamic PPIN	Yes	Temporal split	Temporal validation	Accuracy, precision, recall	Acc: 60.0%; Pre: 82.0%; Rec: 50.0%	Time-aware interaction learning	Limited recall
Li et al. (2024) [115]	Dynamic PPIN reconstruction	DIP temporal dataset	Yes	Temporal split	Reconstruction evaluation	Precision, recall	Precision: 73.0%; recall: 71.0%	Dynamic network reconstruction	Limited benchmark standardization
He et al. (2025) [116]	Dynamic PPIN reconstruction	Babu dataset	Yes	Independent test set	Independent testing	Acc, Pre, Rec, MCC	Acc: 91.0%; MCC: 84.0%	Temporal network reconstruction	Limited biological validation

Abbreviations: AAC, Amino Acid Composition; AC, Autocovariance; AUC, Area Under the Receiver Operating Characteristic Curve; AUPR, Area Under the Precision–Recall Curve; CNN, Convolutional Neural Network; CV, Cross-Validation; DHT, Discrete Hartley Transform; DL, Deep Learning; GAT, Graph Attention Network; MCC, Matthews Correlation Coefficient; PPI, Protein–Protein Interaction; PPIN, Protein–Protein Interaction Network; RF, Random Forest; ROC-AUC, Receiver Operating Characteristic Area Under the Curve; RPI, RNA–Protein Interaction; SVM, Support Vector Machine; VGAE, Variational Graph Autoencoder.

## Data Availability

The data supporting the findings of this article are included within the manuscript.
